# Construction of a cancer-perturbed protein-protein interaction network for discovery of apoptosis drug targets

**DOI:** 10.1186/1752-0509-2-56

**Published:** 2008-06-30

**Authors:** Liang-Hui Chu, Bor-Sen Chen

**Affiliations:** 1 Lab of Control and Systems Biology, National Tsing Hua University, Hsinchu, 300, Taiwan

## Abstract

**Background:**

Cancer is caused by genetic abnormalities, such as mutations of oncogenes or tumor suppressor genes, which alter downstream signal transduction pathways and protein-protein interactions. Comparisons of the interactions of proteins in cancerous and normal cells can shed light on the mechanisms of carcinogenesis.

**Results:**

We constructed initial networks of protein-protein interactions involved in the apoptosis of cancerous and normal cells by use of two human yeast two-hybrid data sets and four online databases. Next, we applied a nonlinear stochastic model, maximum likelihood parameter estimation, and Akaike Information Criteria (AIC) to eliminate false-positive protein-protein interactions in our initial protein interaction networks by use of microarray data. Comparisons of the networks of apoptosis in HeLa (human cervical carcinoma) cells and in normal primary lung fibroblasts provided insight into the mechanism of apoptosis and allowed identification of potential drug targets. The potential targets include BCL2, caspase-3 and TP53. Our comparison of cancerous and normal cells also allowed derivation of several party hubs and date hubs in the human protein-protein interaction networks involved in caspase activation.

**Conclusion:**

Our method allows identification of cancer-perturbed protein-protein interactions involved in apoptosis and identification of potential molecular targets for development of anti-cancer drugs.

## Background

Study of interactome, the entire set of molecular interactions within cells, has provided many insights into the etiology and regulation of cancer [1,2]. Tumorigenesis is a multi-step process caused by genetic alterations that drive the progressive transformation of normal cells into malignant cells. At the molecular level, genetic mutations, translocations, amplifications, deletions, and viral gene insertions can alter translated proteins and thereby disrupt signal transduction pathways and protein-protein interactions that are essential for apoptosis and other important cellular processes [3]. Inactivation of pro-apoptotic proteins or up-regulation of anti-apoptotic proteins results in unchecked growth of cells and ultimately to cancer [4]. From a systems biology perspective, cancer is mainly caused by malfunctions of perturbed protein interaction networks in the cell [5,6].

Apoptosis is necessary for normal human development and survival, in that cells must die in order to prevent uncontrolled growth [7]. Apoptosis requires activation of multiple pathways via regulated protein-protein interactions [8]. Apoptosis is mediated by an intrinsic pathway, which is triggered by "death stimuli" (e.g., DNA damage, oncogene activation, among others) within a cell, or by an extrinsic pathway, which is initiated by binding of an extracellular "death ligand". The extrinsic pathway can link to the intrinsic pathway, which then triggers the release of mitochondria proteins via protein-protein interactions [4,8,9]. During apoptosis, several proteins are released from the intermembrane space of the mitochondria into the cytoplasm and these proteins activate initiator caspases and trigger a series of protein-protein interactions in the caspase cascade. Evading apoptosis is one of the six acquired capabilities of cancer cells [3], and anticancer treatment using cytotoxic drugs is considered to mediate cell death by activating key elements of the apoptosis program and the cellular stress response [10]. Comprehensive knowledge of protein-protein interactions provides a framework for understanding the biology of cancer as an integrated system [11].

Most gene products mediate their functions within complex networks of interconnected macromolecules, forming a dynamic topological interactome [11,12]. High throughput two-hybrid experiments [13,14] and several online interactome databases, such as BIND [15], HPRD [16], Intact [17], and Himap [18], allow analysis of the global topologies of human protein-protein interactions. BIND [15] is a database designed to store full descriptions of interactions, molecular complexes and pathways. HPRD [16] provides detailed data including protein sequences, localization, domains, and motifs, and thousands of protein-protein interactions, with other data. Intact [17] contains an enrichment of protein-protein interactions, related literature, and experimental detail. Himap [18] combines two datasets of yeast-two-hybrid experiments [13,14] to form a human protein reference database [16], with references to functions and predictions.

However, experimental and database approaches often yield "false-positives" [19]. For example, yeast two-hybrid experiments based on transactivation of reporter genes require the presence of auto-activators, where the bait activates gene expression in the absence of any prey [11]. The yeast two-hybrid technique can yield false-positives (spurious interactions detected because of the high-throughput nature of the screening process), and false-negatives (undetected interactions) [19,20]. Computational methods can refine protein-protein interaction networks and result in fewer false-positives [21,22]. Because of the complex nature of interactomes, such as that observed in the apoptosome complex during caspase formation [7,8,23], a nonlinear mathematical model provides better characterization than a linear model [24,25]. In addition, a stochastic model allows consideration of intrinsic and extrinsic molecular "noise" that causes stochastic variations in transcription and translation [24]. In this paper, we describe a nonlinear stochastic model that characterizes dynamic protein-protein interaction networks of apoptosis in cancerous and normal cells.

In this study, we built an initial protein-protein interaction network based on two human yeast two-hybrid data sets [13,14] and four online interactome databases such as BIND [15], HPRD [16], Intact [17], and Himap [18]. Next, we constructed a nonlinear stochastic model of dynamic protein-protein interactions to eliminate false-positives from the network by applying a statistical method (Akaike Information Criterion, AIC) to the high-throughput protein interaction data. We regard all proteins in an organism as a large dynamic interaction system. Protein-protein interactions are considered as nonlinear stochastic processes with several expression profiles of interactive protein partners as input, and the expression profile of a target protein as output. Because of random noise and uncertainties during experiments, we describe protein-protein interactions with stochastic discrete nonlinear dynamic equations. We considered linear individual (or binary) protein interactions and nonlinear cooperative protein complex interactions, but not DNA-protein or metabolite interactions. First, we constructed protein-protein interaction networks of apoptosis in HeLa (human cervical carcinoma) cells and normal primary human lung fibroblasts based on microarray data [26]. Next, we obtained the cancer-perturbed protein-protein interaction network by comparison of apoptosis in normal cells via gain-of-function and loss-of-function networks. Because current drugs designed to induce apoptosis kill cancer cells as well as normal cells, these cancer-perturbed protein-protein interaction networks allow identification of potential selective targets of apoptosis-promoting drugs [5].

## Results and Discussion

### Construction of the cancer-perturbed protein-protein interaction network of apoptosis

Initially, we selected proteins that are known to have roles in apoptosis and considered them as the "core nodes" of our network. These included BAX (BCL2-associated X protein), BCL2 (B-cell CLL/lymphoma 2), BID (BH3 interacting domain death agonist), CASP3 (caspase-3), BIRC4 (baculoviral IAP repeat-containing 4), CASP9 (caspase-9), CYCS (cytochrome *c*, somatic), and DIABLO (diablo homolog, Drosophila). Networks, such as ours, that are developed from initially selected genes or proteins as the core nodes are referred to as "BRAC-centered networks" [27]. Our initial apoptosis network contained 207 protein nodes and 841 protein-protein interaction edges.

**Figure 1:**
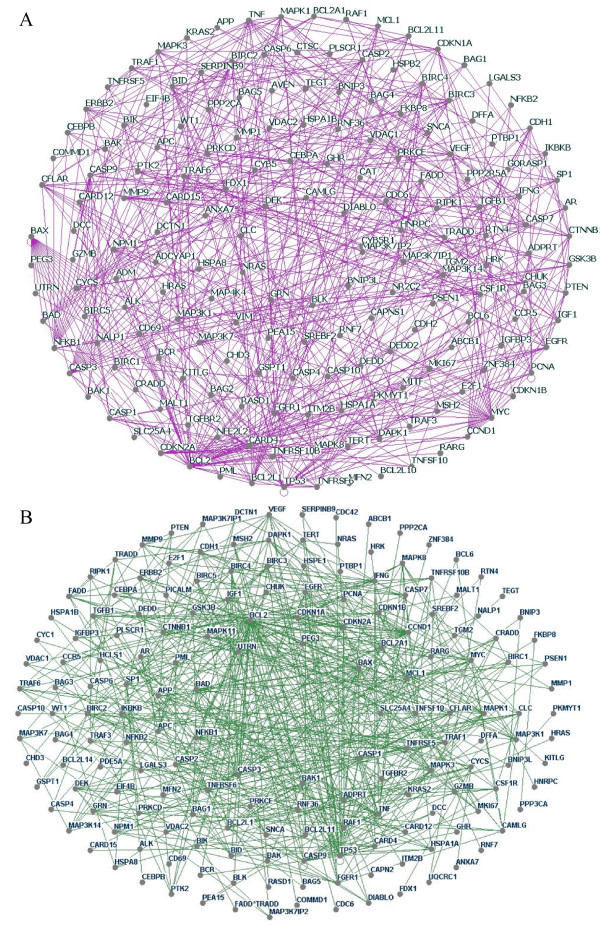
**Global protein-protein interactions of apoptosis in cancerous and normal cells**. (A) Apoptotic protein-protein interaction network in HeLa cells, showing 183 nodes and 552 edges. (B) Apoptotic protein-protein interaction network in normal primary lung fibroblasts, showing 175 nodes and 547 edges. Each interaction was calculated twice and only interactions with two '1' scores after AIC evaluation was considered 'true' interactions (see Supplementary Table 1 for detailed information). All protein-protein interaction networks in this study were constructed with Osprey version 1.2.0.

From equations (1) to (13) (see "Methods"), we calculated each protein interaction twice, with each partner considered as the target protein. The final results are shown in Figure 1 and Supplementary Table 1 (see "Additional file 1"). Figure 1 illustrates the individual and cooperative protein-protein interaction networks of apoptosis in cancerous cells (183 nodes and 552 edges) and normal cells (175 nodes and 547 edges). These networks are easily modeled by undirected graphs, where the nodes are proteins and two nodes are connected by an undirected edge if the corresponding proteins bind one another [28].

**Figure 2:**
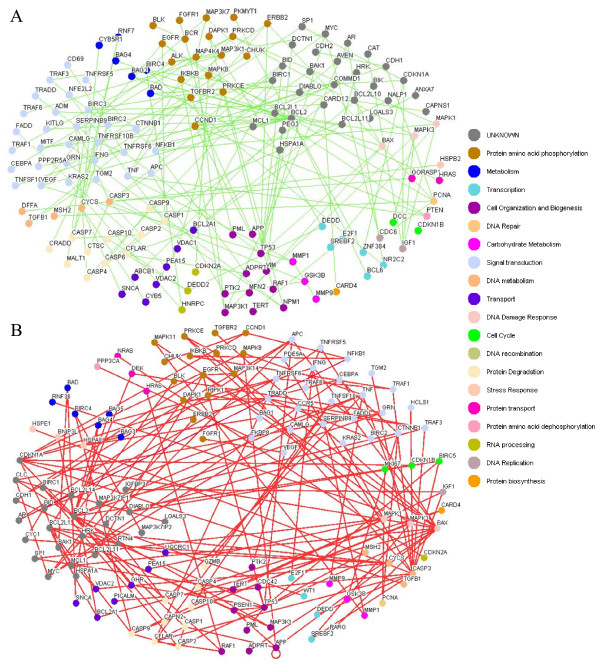
**Cancer-perturbed protein-protein interactions in the apoptosis network**. (A) 'Gain-of-function' network, showing 140 nodes and 157 edges. (B) 'Loss-of-function' network, showing 126 nodes and 162 edges. Colors of nodes represent Gene Ontology annotations. Supplementary Tables 2 and 3 list proteins with detailed Gene Ontology annotations, with ranking according to the degree of perturbation.

Supplementary Table 1 compares the networks of apoptosis in HeLa cells and normal primary human lung fibroblasts. These data show, for example, that BAX and PEG3 interact in both cell types, that BAX and CCND1 interact in neither cell type, that BAX and RARG interact in normal cells but not in cancerous cells, and that BAX and BCL2L1 interact in cancerous cells but not in normal cells. In order to identify drug targets for anti-cancer drugs, it is important to identify cancer-perturbed protein-protein interaction networks to identify drug targets to kill cancer cells [3]. If an interaction is absent in normal cells, but present in cancer cells, we call it "gain-of-function"; if an interaction is present in normal cells but not in cancerous cells, we term it "loss-of-function". For the 841 interactions that we identified, we classified 157 (18.7%) as "gain-of-function" and 162 (19.3%) as "loss-of-function" (Figs 2A and 2B). This network analysis identified 38% (18.7%+19.3%) of all protein-protein interactions during apoptosis as potential drug targets.

Figure 2 shows nodes that are colored according to protein family, as annotated by the Gene Ontology (GO) hierarchy, and illustrates gain- and loss-of-function interactions derived from Supplementary Tables 2 and 3 (see 'Additional file 2' and 'Additional file 3'). Proteins are listed with Gene Ontology (GO) annotations according to the number of perturbed interactions. All protein candidates with more than five degrees of perturbations are shown, to illustrate the number of links with perturbed nodes. Gain-of-function proteins with more than five degrees of perturbations in Figure 2A include BCL2, CASP3, TP53, BCL2L1, PRKCD, MAPK3, NFKB1, BIRC3, CCND1, and PCNA. Loss-of-function proteins with more than five degrees of perturbations in Figure 2B include BCL2, BAX, CASP3, CDKN1A, TP53, BCL2L1, TNF, CASP9, EGFR, MAPK1, APC, TNFRSF6, BAK1, MYC, CFLAR, and APP (see Supplementary Tables 2 and 3). The BCL2 protein has the highest degree of perturbations (18 and 17) in cancerous and normal cells, respectively.

In order to confirm the topology of our networks (Fig. 2), we calculated the false-positive and false-negative rates for the 86 BCL2-interacting proteins in normal cells by use of the HPRD (Human Protein Reference Database) [16] and literature review (see the representative example in Supplementary Table 4 from 'Additional file 4'). After refinement by our algorithms, we reduced the false-positive rate to 1.16%. However, the false-negative rate remained at 41.87%, indicating incomplete construction of the network from current experiments and databases. Therefore, compensation by *k *(see equation (1) in "Methods") is important for estimation of model parameters.

### Cancer-perturbed apoptosis mechanism at the systems level

In many cancers, pro-apoptotic proteins are inactivated or anti-apoptotic proteins are upregulated, leading to unchecked growth and an inability to respond to cellular stresses [4]. These gain- and loss-of-function mutations lead to aberrations in protein-protein interaction networks. An integration of interactome data and genomic data can provide a clearer understanding of the functional relationships that underlie apoptosis and other biological processes [11,21]. The results of our investigation of the apoptosis mechanism at the systems level and the elucidation of cancer-perturbed protein-protein interaction network topology are depicted in Figs. 2A and 2B.

#### Extrinsic pathway, intrinsic pathway and crosstalk

Members of the death receptor superfamily trigger the extrinsic apoptosis pathway upon recruit of caspase-8 through the adaptor protein FAS (TNFRSF6)-associated death domain (FADD) [7]. Binding of a "death ligand" to a receptor triggers formation of a signaling complex that activates caspase-8 or caspase-10, which then activates caspase-3, and finally promotes cell death [23]. Extracellular and intracellular stress triggers the intrinsic apoptosis pathway (mitochondria pathway), which involves activation of pro-apoptotic members of the Bcl-2 family [8]. Bid, a pro-apoptotic member of the Bcl-2 family, allows crosstalk between the extrinsic and intrinsic pathways. The three subfamilies of Bcl-2-related proteins are the anti-apoptotic proteins (e.g., BCL2 and BCL2L1), the pro-apoptotic multi-domain proteins (e.g., BAX and BAK), and pro-apoptotic BH3-only proteins (e.g., BID and BIM) [9,29].

Of the 19 Bcl-2 proteins or regulators (see "goProcess" in Supplementary Tables 2 and 3), 11 are present in Figs. 2A and 2B (BAD, BAG4, BAK1, BAX, BCL2, BCL2A1, BCL2L1, BCL2L11, BID, HRK, and MCL1), 4 are present in Fig. 2A alone (BAG2, BCL2L10, BCL6, and BIK) and 4 are present in Fig. 2B alone (BAG1, BAG3, BAG5, and BCL2L14). This indicates that there is not a simple dichotomy between gain-of-function and loss-of-function proteins in the cancer interactome. For example, BAX has 4 gain-of-function interactions with BCL2L1, TP53, MFN2, and BCL2L10 but it also has 13 loss-of-function interactions with RARG, MCL1, BCL2A1, CDKN1A, MAPK11, APP, APC, CASP2, PRKCE, RNF36, ADPRT, TGFBR2, and TNFRSF5 (Fig. 2).

#### Caspases family and caspases regulators

The apoptosis death signal is activated via a series of protease caspases (initiator caspases-2, -8, -9, and -10, and effector caspases-3, -6, and -7) which require activation by proteolysis [8,9]. With the exception of CASP6, which is present only in the gain-of-function network, the caspases (including CASP1, CASP2, CASP4, CASP7, CASP9 and CASP10) occur in the gain-of-function and loss-of-function networks. Seven caspase activators, inhibitors, and regulators (including BAX, TP53, CFLAR, CYCS, CARD4, BIRC4, and DIABLO) are present in both networks (Figs. 2A and 2B). This reveals the different roles of caspase regulators in cancerous and normal cells.

#### Regulation of apoptosis at the systems level

Besides Bcl-2 and caspases, proteins involved in apoptosis regulation include BIRC3, PTEN, CARD12, MAP3K7, DEDD2, MITF, MALT1, BCL6, NALP1, CRADD, RTN4, PSEN1, IGFBP3, BNIP3L, RARG, CFLAR, TRAF3, TRAF1, MCL1, CARD4, TRAF6, VEGF, BIRC2, FGFR1, PEA15, DEDD, MMP9, HRK, and TP53 (Figs. 2A and 2B). Thus, we have identified 29 proteins, in addition to members of the Bcl-2 and caspase families, that regulate apoptosis at the systems level.

#### Apoptosis and cell cycle regulation

Proteins generally function as components of complexes that contain other macromolecules, to carry out specific biological processes. Networks of interactions connect different cellular processes [11]. We have identified 30 proteins in the protein-protein interaction network of apoptosis that also participate in cell-cycle regulation. These are PTEN, CDC6, MFN2, PKMYT1, DCC, and E2F1 in Fig. 2A, and BIRC5, NRAS, MKI67, E1F1, PPP3CA, BCL2, TP53, MAPK3, CCND1, PCNA, EGFR, MAPK1, VEGF, BAX, CDKN1A, TGFB1, APC, MSH2, APP, KRAS2, HRAS, CDKN1B, CDKN2A, and PML in Fig. 2B.

Although Figs. 2A and 2B describe the perturbation of apoptosis at the systems level, most proteins with high degree of perturbation (Fig. 2A) are also included in Fig. 2B. In other words, it is not possible to uniquely describe protein hubs as "gain-of-function" or "loss-of-function" hubs. Therefore, we summarized the degree of perturbation (Supplementary Tables 2 and 3) to identify the perturbed protein hubs in the network of cancer cells. After identifying targeted proteins as inhibitors or activators, it is necessary to study how a drug target is wired into the control circuitry of a complex cellular network [30]. Next, we show a flow chart for identification and prediction of apoptosis drug targets in cancer drug discovery.

### Prediction of apoptosis drug targets using cancer-perturbed networks of apoptosis

**Figure 3:**
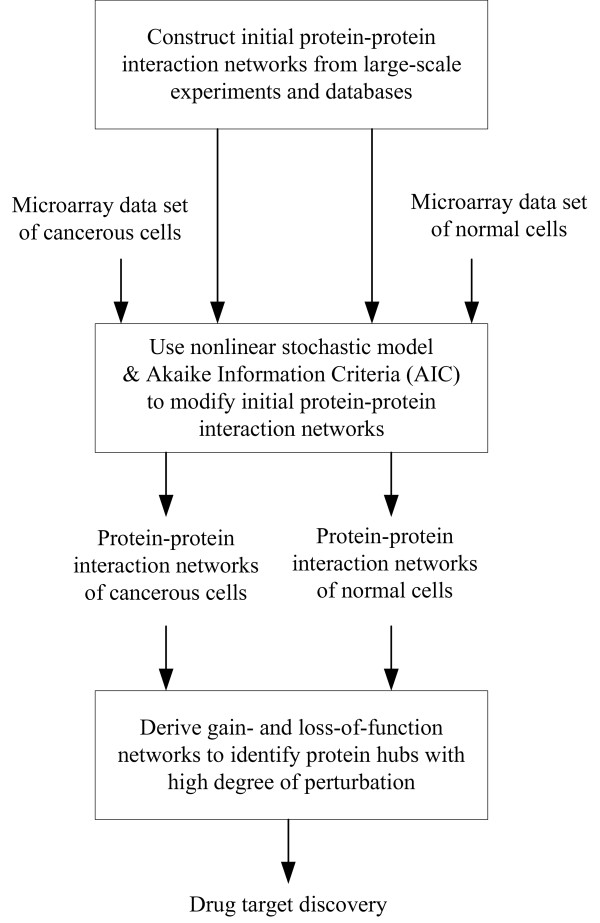
Flow chart for identification of potential drug targets in the cancer-perturbed network using microarray data.

Systems-based drug design is a major application of systems biology [5,25,31]. This method constructs disease-perturbed protein-protein interaction networks and identifies potential drug targets by comparison of the networks of normal and abnormal cells. This contrasts with the traditional approach, which reduces cellular processes to their individual components or signal transduction pathways and targets a specific molecule or signaling pathway. A limitation of this traditional approach is that a single molecule or pathway does not adequately describe most biological systems, including those affected in cancer [25]. By comparisons of the protein-protein interaction networks of normal and cancerous cells derived from microarray data, we can identify potential drug targets through a systems-based approach (Fig. 3).

Thus, we built initial protein-protein interaction networks from large-scale experiments and databases, and then employed each microarray data set of HeLa cells and primary lung fibroblasts to modify these networks. For network modification, we used a nonlinear stochastic model and the Akaike Information Criterion (AIC). We next compared the networks of cancerous and normal cells, derived gain-of-function and loss-of-function networks, and identified protein hubs with high degree of perturbation as potential drug targets.

Scale-free networks are extremely sensitive to removal of targeted hubs (i.e., attack vulnerability [11,12,32]), so we summed the degree of perturbation (i.e., connectivity) of each node in the cancer-perturbed network (Supplementary Tables 2 and 3) to obtain these perturbed hubs. Proteins with sum of degree of perturbation ≥ 8 (Table 1) differentiate the cancerous and normal interactomes and are potential drug targets [6,25]. We classified the 17 potential drug targets (Table 1) into six categories: (i) Intrinsic pathway: BCL2, BAX, BCL2L1, BID, and CYCS; (ii) Extrinsic pathway: TNF and TNFRSF6; (iii) Common pathway: CASP3 and CASP9; (iv) Apoptosis regulators: TP53, MYC, CFLAR, and EGFR; (v) Stress-induced signaling: MAPK1 and MAPK3; and (vi) Others: CDKN1A and CCND1. Our results indicate that most proteins interact with few partners, whereas hubs interact with many partners, consistent with current views on interactome networks with a scale-free or power law degree distribution [11].

**Table 1:** 17 potential drug targets ranked by sum of degree of perturbation ≥ 8 in Supplementary Tables 2 and 3

Protein targets	Sum of degree of perturbation	Agents	Literature review
BCL2	35	G3139 (Genasense), ABT-737	[4,29,33-36,38]
CASP3	22	Synthetic activators of caspases/Apoptin/IAP targets surviving	[33,34,36]
BAX	17	Gene therapy through Bax vectors	[34]
TP53	17	ONY-015/INGN201/MDM2 inhibitors	[4,34,38,39]
BCL2L1	13	Antisense BCL-xL	[34]
CDKN1A	13		[43]
TNF	10		[4,34]
EGFR	9	Trastuzumab (Herceptin)	[34]
MAPK1	9	CI-1040/PD0325901/ARRY-142886	[41,42]
MAPK3	9	CI-1040/PD0325901/ARRY-142886	[41,42]
MYC	9	Bortezomib (Proteosome Inhibitors)	[40]
BID	8		[34]
CASP9	8	Caspases activators/Apoptin/surviving	[33,34,36]
CCND1	8		[44]
CFLAR	8		[23]
CYCS	8		[37,38]
TNFRSF6	8		[34]

#### Intrinsic pathway: BCL2, BAX, BCL2L1, and CYCS

Defective apoptosis in human cancers often results from over-expression or inhibition of BCL2 proteins. These proteins regulate mitochondrial permeability by inhibiting (e.g., BCL2 and BCL2L1) or promoting (e.g., BAX and BID) release of cytochrome *c *(CYCS) [33]. BCL2 and several anti-apoptotic relatives, such as BCL2L1, associate with the mitochondrial outer membrane and the endoplasmic reticulum nuclear membrane and maintain the integrity of these membranes. Initiation of apoptosis requires pro-apoptotic family members that closely resemble BCL2 and distantly related proteins that are related only by the small BH3 protein-interaction domain [29]. In our results, proteins with gain-of-function interactions with BCL2 include CCND1, BAD, MCL1, MAPK3, ADM, KITLG, EGFR, BAG2, PKMYT1, TP53, PCNA, MITF, ABCB1, BCL6, ZNF384, HRK, PPP2R5A, and VEGF (Supplementary Table 2). Proteins with loss-of-function interactions with BCL2 include CDKN1A, TNF, WT1, BAG4, BCL2L14, DEK, GRN, RAF1, BLK, BAG5, CAPN2, GHR, CDKN1B, RTN4, BNIP3L, MAP3K1, and CLC (Supplementary Table 3). We predict BCL2 to be the best potential drug target because this protein best differentiates protein-protein interaction networks of HeLa and normal cells [5,25].

Our analysis agrees with the conclusions of previous studies which showed that BCL2 protein family members are good targets for cancer therapy. Drugs that target BCL2 include Genasense [4,34] and ABT-737 [35]. The activation of Bax can be induced by gene therapy through delivery of Bax vectors, and this approach has been successful in inducing apoptosis in cancer cell lines [4]. Antisense BCL2L1 (BCL-xL) downregulates the expression of BCL2 and BCL2L1, induces apoptosis, and inhibits growth of several tumor types *in vitro *and *in vivo *[34]. Unfortunately, targeting of BCL-2 also causes adverse effects, presumably because many normal cells depend on proteins in the BCL-2 family to maintain normal mitochondrial function. Difficulty in using BCL-2 antisense DNA or RNA as a delivery system is a problem with Genasense [36]. Cytochrome *c*, once released into the cytosol, interacts with Apaf-1 and this leads to activation of caspase-9 proenzymes [34]. The chief function of BCL-2 proteins is to regulate the release of cytochrome *c *from mitochondria. Thus, targeting CYCS or Apaf-1 would also be expected to cause severe adverse effects [37,38].

#### Extrinsic pathway and crosstalk: TNF, TNFRSF6, and BID

The extrinsic pathway activated by death receptors, such as Fas (TNFRSF6/APO-1/CD95) and other TNF receptor family members, allows apoptosis to maintain normal tissue homeostasis [7]. Although death receptors of the TNF superfamily members are potential targets for anti-cancer drugs, toxic side effects have been observed that place limits on their therapeutic use [4]. TNF and Fas (TNFRSF6) were also found to activate nonspecific TNF receptors resulting in extensive ischemic and hemorrhagic lesions in several tissues leading to septic shock and fulminating hepatic failure in animal models [34].

A more promising approach involves targeting the TRAIL (TNF-Related Apoptosis Inducing Ligand) receptors [4,7,34]. Activation of the TRAIL death receptor can kill cancerous cells but not normal cells, whereas monoclonal antibodies against TRAIL and recombinant TRAIL ligand can cause TRAIL resistance [36]. Thus, administration of such a drug might cause tumors to develop resistance, or cause the death of normal cells. BID, a pro-apoptotic Bcl-2 family member, provides crosstalk and integration between the death-receptor and mitochondrial pathways [8,9]. Targeting BID, however, is rarely discussed, although such work has potential for use in combined therapy [34].

#### Common pathway: CASP3 and CASP9

Caspases are the central components of the apoptotic response network. An effector caspase (e.g., caspase-3) is activated by an initiator caspase (e.g., caspase-9) and the initiator caspase is activated via other protein-protein interactions [8,9]. Targeting inhibitors of caspases could potentially cause apoptosis of cancerous cells. Synthetic activators of caspases include Apoptin and IAP [33,34]. Caspase-3 and -9 are subject to inhibition by IAPs such as Livin [9]. Like BCL-2 inhibitors, XIAP inhibitors must block protein-protein interactions. When released from mitochondria, Smac binds XIAP and inactivates it, triggering apoptosis [36].

#### Apoptosis regulators: TP53, MYC, CFLAR, and EGFR

One of the most dramatic responses to p53 is induction of apoptosis and regulation via the intrinsic pathway [39]. Drug trials that target p53 include gene therapy involving ONYX-015 and INGN201 and antisense therapy that targets a protein controlling p53 activity by Nutlins which blocks p53/MDM2 interaction [4,34]. The proto-oncogene c-MYC encodes a transcription factor that is implicated in various cellular processes, including cell growth, proliferation, loss of differentiation, and apoptosis. The induction of cell-cycle entry sensitizes the cell to apoptosis, so that cell-proliferation and apoptotic pathways are coupled [40]. CFLAR (c-FLIP) regulates caspase-8 and FADD-like apoptosis. Whereas CFLAR blocks the activation of the initiator caspase-8, XIAP can block the initiation phase (by inhibition of caspase-9) and the execution phase (by blocking caspase-3 and caspase-7) [23]. Some agents, for example, agent ZD1839 as an EGFR (epidermal growth factor receptor) inhibitor, do not primarily target apoptosis, but indirectly modulate apoptosis [34].

#### Stress-induced signaling and others: MAPK1, MAPK3, CDKN1A, and CCND1

Proteins of the MAPK (mitogen-activated protein kinase) family are crucial in many signaling pathways [41]. Three MEK (MAPK kinase) inhibitors, CI-1040, PD0325901, and ARRY-142886, are currently in clinical trials for treatment of various cancers [42]. Although some drugs target MAPKs, MAPK1 (ERK or p38), and MAPK3 (ERK1) are not the main drug targets in the apoptotic pathway [34]. CDKN1A (p21) (cyclin-dependent kinase inhibitor-1) plays a role in cell cycle arrest and induction of apoptosis. The activities of cyclin D- and cyclin E-dependent kinases are linked through the Cip/Kip family of Cdk inhibitors, including p27 and p21 [43]. CCND1 is cyclin D1 in the G1/S transition of the cell cycle, and is controlled by the tumor suppressor gene RB through cdk-cyclin D complexes [44].

#### Prediction of additional drug targets by decreasing the degree of perturbation

In addition to identifying several apoptosis drug targets that have already been identified by other studies, we applied our method to predict additional drug targets by decreasing the perturbation threshold. Thus, if we reduce the sum of degree of perturbation to 7, we predict the following additional targets: BAK1, CASP2, BCL2A1, IGF1, PRKCD, NFKB1, and PCNA. NFKB1 has both anti- and pro-apoptotic functions that are determined by the nature of the death stimulus. The drug PS11445 targets the NFKB1 inhibitor IKK*β *[34] but the other proteins have not previously been considered as drug targets.

#### Prediction of new GO annotations of the four proteins: CDKN1A, CCND, PCNA, and PRKCD

If we consider all 24 proteins with sum of degree of perturbation ≥ 7, four proteins (CDKN1A, CCND1, PRKCD and PCNA) are also considered to have a role in apoptosis. If the function of any one protein in a network is known, identification of its interacting partners allows prediction of their functions [11]. However, two of these proteins (PCNA and PRKCD) are known to have a role in DNA damage-induced apoptosis. PCNA (Proliferating Cell Nuclear Antigen) is ubiquitinated and involved in RAD6-dependent DNA repair in response to DNA damage. PRKCD (protein kinase C, delta) is also associated with DNA damage-induced apoptosis [45], whereas gene ontology annotations of PRKCD do not include apoptosis (Supplementary Tables 2 and 3).

Although other methods (such as identification of protein domains) allow prediction of protein-protein interactions in different organisms [46-48], these methods do not allow identification of potential drug targets. Our method provides efficient and precise prediction of anti-cancer drug targets and also specifies these target proteins with detailed Gene Ontology annotations. This will help researchers identify additional drug targets by examination of other cellular mechanisms involved in cancer. Network modeling has been used to identify genes potentially associated with breast cancer in a BRAC-centered network [27]. However, there are very few time-series microarray databases for cancerous and normal cells, so it is difficult to use our method to compare protein hubs for different types of cancer. Moreover, our method does not address the potential adverse effects of targeting a specific protein and the problem of drug delivery. We expect that more genomic time-series microarray experiments and clinical research will address these limitations in the future.

### Caspase activation through static and dynamic hubs

Static network topology is not sufficient to define function, and incorporating time-dependent expression data is important for understanding pathway function [20]. A number of computational approaches have been proposed for prediction of protein-protein interactions, such as domain-domain interactions [46], the confidence score resulting from sequence similarity and number of edges [2], and integration of genomic data sets [49]. A nonlinear stochastic model can depict protein-protein interaction networks at different times by using linear binary interactions and nonlinear protein complex relationships. Previous studies have used linear stochastic models to describe the multiple feedback loops of p53 [50], but nonlinear effects cannot be depicted by this method. Our method also illustrates the dynamic behavior of protein-protein interaction networks, which cannot be examined by probabilistic methods of data integration [49].

**Figure 4:**
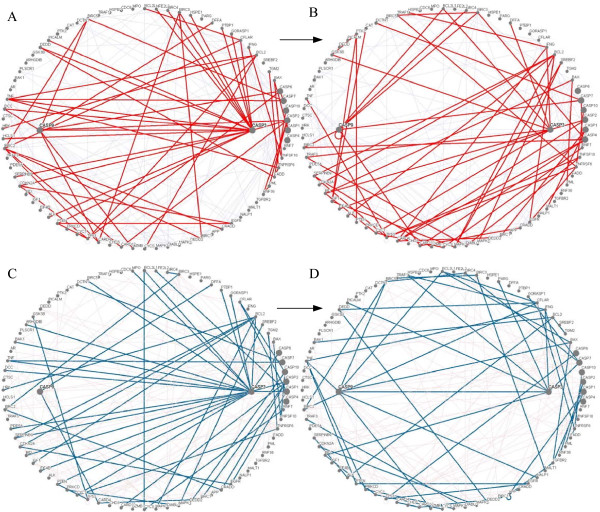
**Dynamic protein-protein interactions in caspase formation**. (A) Protein-protein interactions within the hub caspases of cancer cells during 0–8 hours after induction of apoptosis. Distinct interactions at different times are marked with bold lines. (B) During 4–30 hours after induction of apoptosis in cancer cells. (C) During 0–8 hours after induction of apoptosis in normal cells. (D) During 4–36 hours after induction of apoptosis in normal cells (see Supplementary Tables 5 and 6).

Networks consist of party hubs (static hubs) and date hubs (dynamic hubs). Party hubs are found in static complexes with most of their partners present at the same time, whereas date hubs bind their interaction partners at different times or locations [12,51]. To further investigate dynamic apoptotic properties in human cells, we considered caspases as protein hubs in our dynamic nonlinear stochastic protein-protein models (Figs. 4A–D, Supplementary Tables 5 and 6 in 'Additional file 5' and 'Additional file 6'). In order to identify party hubs and date hubs, we first summed the degree of perturbation of each protein as 'plus degree of perturbation' and then subtracted the degree of perturbation of each protein as 'minus degree of perturbation' at two time periods in cancerous and normal cells. If the plus degree of perturbation of one protein was ≥ 20, we defined this protein as a 'hub'. If the minus degree of perturbation of the hub was ≤ 3, we defined the hub as a party hub (static hub). If the minus degree of perturbation of the hub was >3, we defined the hub as a date hub.

Figures 4A–D illustrate the time-dependence of the networks, where bold lines represent distinct interactions at different times. Caspase signaling results in time-variant protein-protein interactions, and dynamic modeling allows specification of the time-dependent interactome. In cancerous cells, the date hubs include BIRC2, CASP2, and CASP3, and the party hubs include TP53, TNF, BIRC3, BAX, CASP1, and CASP9. In normal cells, date hubs include CASP3 and CASP9 and party hubs include TNFRSF6, TP53, BIRC2, BIRC3, BCL2, BAX, and CASP1. Effector caspase-3 is a date hub in both cell types because intrinsic and extrinsic pathways converge on caspase-3. Because date hubs appear to be more important than party hubs [51], caspase-2 and -9 are important date hubs that differentiate network topologies of cancerous and normal cells. TP53, BIRC3, BAX, and CASP1 are party hubs in both cell types. Party hubs are found in static complexes where they interact with most of their partners simultaneously [51]. In other words, we believe these four proteins play central roles in functional complexes in both cancerous and normal cells.

## Conclusion

The cancer-perturbed protein-protein interaction networks of apoptosis that we developed here shed light on the mechanism of cancer at the systems level and allow identification of potential drug targets. In this study, we applied nonlinear stochastic modeling to describe individual and cooperative protein interactions. Our method is more precise than the linear models used in previous research. We successfully integrated microarray and proteome databases to identify cancer-perturbed protein-protein interaction networks. Our predictions of potential drug targets agreed with potential targets identified by other studies and also identified additional targets which may guide the development of new anti-cancer drugs in future. We also identified static and dynamic hubs in human protein-protein interaction networks, which have heretofore been identified only in yeast.

## Methods

### Construction of initial protein-protein interaction networks

A comprehensive understanding of protein-protein interactions in an organism provides a framework for understanding biology as an integrated system, and human perturbed protein-protein interaction networks offer insight into disease mechanisms such as cancer at the systems level [5,11,25]. Before construction of cancer-perturbed protein-protein interaction networks to explore their roles in the mechanism of cancer, protein-protein interaction networks for both cancer and normal cells must be constructed for comparison. The systematic experimental mappings of the human interactome include yeast two-hybrid systems [13,14], which reveal thousands of preys and baits in the protein matrix. Several online databases such as BIND [15], HPRD [16], Intact [17], and Himap [18] provide fundamental global topologies of human protein-protein interactions. The combined use of these databases [15-18] assists precise estimation of parameters as the basis of construction of protein-protein interaction networks, because incomplete rough networks can lead to biased or possibly erroneous conclusions [11]. We first constructed an initial protein-protein interaction network using two yeast-two-hybrid experiments and four online databases.

Studies of large-scale protein-protein interactions allow development of protein interaction networks, but all large-scale experiments and databases contain high rates of false-positives [52]. Previous studies have demonstrated that use of multiple functional databases allows better identification of protein-protein interactions and leads to better prediction of the function of unknown proteins [49]. Therefore, we integrated different databases and experiments to refine our network [19]. After development of our initial network, we used microarray data to remove false-positive interactions.

### Selecting and processing experimental data

We used microarray data [26] to compare gene expression in HeLa cells and normal primary human lung fibroblasts subjected to several stresses (e.g., heat shock from 37°C to 42°C, oxidative stress with menadione or hydrogen peroxide, and endoplasmic reticulum stress with DTT (dithiothreitol) or tunicamycin). Gene expression in HeLa cells and normal fibroblasts, both of which were treated with 2.5 mM DTT, were our microarray data sources. Gene expression was recorded at 0–30 h after stress in HeLa cells and 0–36 h in normal cells [26] (Supplementary Tables 7 and 8; see 'Additional file 7' and 'Additional file 8').

### Nonlinear stochastic interaction model of initial networks

We used a nonlinear stochastic model to mimic the dynamic interactions among proteins to remove false-positives and refine the network. In recent years, some systems and computational biologists employ a dynamic perspective to describe biological functions, because of their dynamic nature [53-55]. We considered two types of interactions, binary protein interactions and protein complex interactions, but not DNA-protein or metabolite interactions. We denote these basal interactions as *k *in our model, whereas *ε*[*t*] represents stochastic molecular events, such as molecular fluctuations of the target protein.

**Figure 5:**
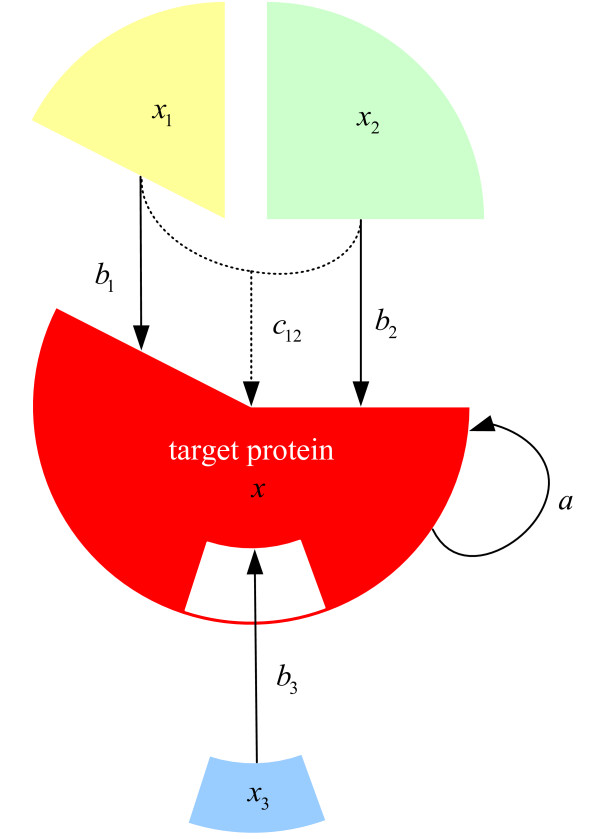
**Graphical representation of individual protein interactions and cooperative protein interactions**. Our dynamic protein interaction equation includes three individual or binary protein-protein interactions to the target protein *x *[*t*] (protein *x*_1 _[*t*], *x*_2 _[*t*], and *x*_3 _[*t*]) and one cooperative interaction between protein *x*_1 _[*t*] and *x*_2 _[*t*] to the target protein *x *[*t*]. *a *denotes the influence of a target protein at one time point to the target protein at the next time point; *b*_*i *_denotes individual or binary interaction of protein *i *with target protein *x *[*t*]; and *c*_*ij *_denotes the cooperative interaction ability of protein *i *and protein *j *on the target protein *x*.

In this study, we define 'individual protein-protein interactions' as binary protein-protein interactions, and 'cooperative interactions' as the interaction of a protein complex with the target protein. *x* [*t*] denotes the expression profile of target protein *x *at time point *t*; *a *denotes the influence of a target protein at one time point to the target protein at the next time point; *b*_*i *_denotes individual or binary interaction of protein *i *with target protein *x *[*t*]; and *c*_*ij *_denotes the cooperative interaction ability of protein *i *and protein *j *on the target protein *x*. Thus, Fig. 5 shows three individual or binary protein-protein interactions to the target protein *x *[*t*] (protein *x*_1 _[*t*], *x*_2 _[*t*], and *x*_3 _[*t*]) and one cooperative interaction between protein *x*_1 _[*t*] and *x*_2 _[*t*] to the target protein *x *[*t*]. By consideration of basal interactions *k *and stochastic events *ε*[*t*], we can express the dynamic model of the target protein time profile *x *[*t*] as: *x *[*t *+ 1] = *ax *[*t*] +*b*_1_*x*_1 _[*t*] +*b*_2_*x*_2 _[*t*] + *b*_3_*x*_3 _[*t*] + *c*_12_*x*_12 _[*t*] + *k + ε *[*t*]. This equation is based on a previously developed model [56].

Therefore, dynamic interactions between upstream proteins and their targets can be written as:

(1) $$x[t+1]=ax[t]+\sum_{i=1}^{N}b_{i}x_{i}[t]+\sum_{i=1}^{N}\sum_{j=1}^{N}c_{ij}x_{ij}[t]+k+\varepsilon [t]$$

This equation is based on a previously developed model [56-58].

*Remark*: The discrete-time dynamic model in equation (1) is based on the discrete sampling of the following continuous differential model:

$$\dot{x}[t]=-\lambda x[t]+\sum_{i=1}^{N}b_{i}x_{i}[t]+\sum_{i=1}^{N}\sum_{j=1}^{N}c_{ij}x_{ij}[t]+k+\varepsilon [t],$$

where $\dot{x}(t)=\frac{dx(t)}{dt}$ denotes the differential of *x*(*t*) with respect to the continuous time *t*, and *λ *denotes the decay rate of *x*[*t*]. By unit sampling and with $\dot{x}[t]$ ≅ *x*[*t *+ 1] - *x*[*t*], we get the following discrete model [59]:

$$x[t+1]-x[t]=-\lambda x[t]+\sum_{i=1}^{N}b_{i}x_{i}[t]+\sum_{i=1}^{N}\sum_{j=1}^{N}c_{ij}x_{ij}[t]+k+\varepsilon [t]$$

or

$$x[t+1]=(1-\lambda )x[t]+\sum_{i=1}^{N}b_{i}x_{i}[t]+\sum_{i=1}^{N}\sum_{j=1}^{N}c_{ij}x_{ij}[t]+k+\varepsilon [t]=ax[t]+\sum_{i=1}^{N}b_{i}x_{i}[t]+\sum_{i=1}^{N}\sum_{j=1}^{N}c_{ij}x_{ij}[t]+k+\varepsilon [t]$$

where *a *denotes the influence of *x *[*t*] on *x *[*t *+1] and is dependent of the decay rate, *λ*.

By iteration of equation (1), we can construct the whole protein-protein interaction network, which is interconnected through the interactions of $\sum_{i=1}^{N}b_{i}x_{i}(t)$ and $\sum_{i}^{N}\sum_{j=1}^{N}c_{ij}x_{ij}(t)$ in equation (1) for all proteins. In equation (1), *x *[*t*] represents the expression profile of the target protein at time point *t*, which is estimated from mRNA expression profiles via a translational sigmoid function [57,58]:

(2) $$x[t]=f(y[t])=\frac{1}{1+\exp[-r(y[t]-M)]}$$

In equation (2), *r *denotes the transition rate of the sigmoid function, and *M *denotes the mean level of mRNA expression of the corresponding protein. $\sum_{i=1}^{N}x_{i}[t]$ denotes all possible individual interactive functions (i.e., all possible binary protein-protein interactions) of *N *interactive protein candidates of the target protein in the initial protein-protein interaction network, *a *denotes the influence of the present target protein on the target protein at the next time point, and *b_i_* indicates the individual interactive ability of protein *i *to target protein *x *[*t*]. $\sum_{i=1}^{N}\sum_{j=1}^{N}c_{ij}x_{ij}[t]$ denotes all possible interactive functions of cooperative protein partners with the target protein in the initial network, where *x*_*ij *_[*t*] denotes nonlinear cooperative interaction between protein *x*_*i *_and protein *x*_*j *_on the target protein, that is, *x*_*ij *_[*t*] = *f *(*y*_*i *_[*t*]) · *f *(*y*_*j *_[*t*]), and *c*_*ij *_denotes the cooperative interaction ability of protein *i *and protein *j *on the target protein (see Fig. 5). We obtained all possible cooperative interactions $\sum_{i=1}^{N}\sum_{j=1}^{N}c_{ij}x_{ij}[t]$ from online high-throughput protein interaction databases that contained putative protein complexes within the network. If the multi-protein complex is composed of three or more proteins, we added all combinations of two cooperative proteins from the complex to determine the nonlinear property because one extreme value in the equation will cause significant aberration in other estimated parameters. The basal interaction *k *in equation (1) represents unknown protein-protein interactions that result from other possible interacting proteins or other influences (e.g., mRNA-protein interactions, protein synthesis). *ε*[*t*] represents random noise arising from model uncertainty and molecular fluctuations of protein interactions with the target protein [24].

With the nonlinear stochastic interaction model (equation 1), we can identify interaction parameters *a*, *b*_*i*_, *c*_*ij *_and *k *using microarray data. After having identified all the interactions with the target protein, upstream proteins can be considered as target proteins, and by repeating this procedure we can identify all interactions of the initial network. If the estimated interaction parameters $\hat{a}$, ${\hat{b}}_{i}$, ${\hat{c}}_{ij}$ and $\hat{k}$ are not significant according to the AIC, the corresponding interactions will be eliminated from the initial network.

### Identification of interactions in the initial protein-protein interaction network

Before modification of the initial network, we estimated interaction parameters of the initial network by use of maximum likelihood estimation. This represents interactions of all possible protein candidates in the initial network. After further rearrangement, equation (1) can be rewritten as:

(3) $$\begin{array}{c} x[t+1]=[\begin{array}{cccccccc} x[t] & x_{1}[t] & \cdots & x_{N}[t] & x_{12}[t] & \cdots & x_{(N-1)N}[t] & 1 \end{array}]\cdot \left[\begin{array}{c} a\\ b_{1}\\ \vdots \\ b_{N}\\ c_{12}\\ \vdots \\ c_{(N-1)N}\\ k \end{array}\right]+\varepsilon [t]\\ \equiv \varphi [t]\cdot \theta +\varepsilon [t] \end{array}$$

where *φ*[*t*] denotes the regression vector composed of many elements that represent expression levels of protein candidates in the initial network at time point *t*.

Employing the cubic spline method [54,58] to interpolate microarray data allows us to obtain as many data points as needed. In general, the number of data points should be at least 5 times the number of parameters to be estimated. The cubic spline method allows us to obtain these values: {*x *[*t*] *x*_*i *_[*t*_*l*_] *x*_*j *_[*t*_*l*_]} for *l *∈ {1 2 ... *M*} and *i ∈ *{1 2 ... *N*}, *j *∈ {1 2 ... *S*}, where *M *denotes the number of microarray data points, *N *denotes the number of possible protein interactions for the target protein, and *S *denotes the number of protein complexes in the network. These data points are used as the basis in our regression vector *φ*[*t*]. Computation of equation (3) at different time points allows us to construct the following vector-form equation:

(4) $$\left[\begin{array}{c} x[t_{2}]\\ x[t_{3}]\\ \vdots \\ x[t_{M-1}]\\ x[t_{M}] \end{array}\right]=\left[\begin{array}{c} \varphi [t_{1}]\\ \varphi [t_{2}]\\ \vdots \\ \varphi [t_{M-2}]\\ \varphi [t_{M-1}] \end{array}\right]\cdot \theta +\left[\begin{array}{c} \varepsilon [t_{1}]\\ \varepsilon [t_{2}]\\ \vdots \\ \varepsilon [t_{M-2}]\\ \varepsilon [t_{M-1}] \end{array}\right]$$

For simplicity, we represent this as:

(5) X = Φ · *θ *+ *ν *

In equation (4), random noise *ε*[*t*_*k*_] is regarded as a white Gaussian noise with zero mean and unknown variance *σ*^2^, that is, *E*{*ν*} = 0, and Σ_*ν *_= *E*{*νν*^*T*^} = *σ*^2^*I*. Next, we employed a maximum likelihood estimation method [59] to estimate *θ *and *σ*^2 ^using regression data obtained from the microarray data of the target protein and proteins with which it interacts. Under the assumption that *ν *is a Gaussian noise vector with *M *– 1 elements, its probability density function is given as follows.

(6) $$p(\nu )={\left({\left(2\pi \right)}^{M-1}\det\Sigma_{\nu }\right)}^{-1/2}\exp(-\frac{1}{2}\nu^{T}\Sigma_{\nu }^{-1}\nu )$$

Since *ν *= X - Φ · *θ *(equation 5), we can rewrite equation (6) as:

(7) $$p(\theta ,\sigma^{2})={\left(2\pi \sigma^{2}\right)}^{-(M-1)/2}\exp\{-\frac{{\left(\text{X}-\Phi \cdot \theta \right)}^{T}(\text{X}-\Phi \cdot \theta )}{2\sigma^{2}}\}$$

Maximum likelihood parameter estimation involves finding *θ *and *σ*^2 ^to maximize the likelihood function in equation (7). In order to simplify the computation, it is practical to take the logarithm of equation (7), which yields the following log-likelihood function:

(8) $$\log L(\theta ,\sigma^{2})=-\frac{M-1}{2}\log(2\pi \sigma^{2})-\frac{1}{2\sigma^{2}}\sum_{k=1}^{M-1}{\left[x[t_{k}]-\varphi [t_{k}]\cdot \theta \right]}^{2}$$

In equation (8), *x *[*t*_*k*_] and *φ*[*t*_*k*_] are the *k*th elements of X and Φ respectively.

Here, we expect the log-likelihood function to have the maxima at *θ *= $\hat{\theta }$ and *σ*^2 ^= ${\hat{\sigma }}^{2}$. The necessary conditions for determining maximum likelihood estimates $\hat{\theta }$ and ${\hat{\sigma }}^{2}$ must consist of [59]:

(9) $$\begin{array}{l} \frac{\partial \log L(\theta ,\sigma^{2})}{\partial \theta }=0\\ \frac{\partial \log L(\theta ,\sigma^{2})}{\partial \sigma^{2}}=0 \end{array}$$

After some computational arrangements from equation (9), the estimated parameters $\hat{\theta }$ and ${\hat{\sigma }}^{2}$ are:

(10) $$\hat{\theta }={\left(\Phi^{T}\Phi \right)}^{-1}\Phi^{T}X$$

(11) $${\hat{\sigma }}^{2}=\frac{1}{M-1}\sum_{k=1}^{M-1}\left[x[t_{k}]-\varphi [t_{k}\right]\cdot \hat{\theta }]=\frac{1}{M-1}{\left(\text{X}-\Phi \cdot \hat{\theta }\right)}^{T}(\text{X}-\Phi \cdot \hat{\theta })$$

After obtaining estimate $\hat{\theta }$, we can rewrite estimated protein-protein interaction (equation 1) as follows:

(12) $$x[t+1]=\hat{a}x[t]+\sum_{i=1}^{N}{\hat{b}}_{i}x_{i}[t]+\sum_{i=1}^{N}\sum_{j=1}^{N}{\hat{c}}_{ij}x_{ij}[t]+\hat{k}$$

We quantified the interactions of all candidate proteins by the process described above.

In equation (12), the estimated parameter $\hat{a}$ denotes the estimation of the residual of target protein, ${\hat{b}}_{i}$ denotes individual interactive rate or binary protein-protein interaction between protein *i *and the target protein, and ${\hat{c}}_{ij}$ denotes cooperative interactive rate or protein complex membership between protein *i *and protein *j*, i.e. protein complex *ij*, to the target protein. A positive value implies positive interaction, a negative value implies negative interaction, and the interactions are more likely as the parameters get larger.

### Modification of initial protein-protein interaction networks

Although our maximum likelihood estimation method allows quantification of all possible interactions of a target protein, we still do not know the significance of the interaction and whether it can be regarded as a true interaction. Thus, we used a statistical approach that involves model validation to evaluate significance levels and refine the network. In this procedure, we employed the Akaike Information Criterion (AIC) to validate the model order or the number of model parameters of the network [59].

The Akaike Information Criterion (AIC) considers estimated residual variance and model complexity as one statistic and provides a measure of the information lost when a model is used. In other words, the AIC is an operational way of trading off the complexity of an estimated model against how well the model fits the data. AIC decreases as residual variance ${\hat{\sigma }}^{2}$ decreases and increases as the number of parameters *p *increases. As the expected residual variance decreases with increasing *P *for inadequate model complexities, there should be a minimum near the correct number of interaction parameters *P*. For a protein interaction model with *P *interaction parameters to fit with data from N samples, the Akaike Information Criterion (AIC) can be written as follows [59]

(13) $$AIC(p)=\log(\frac{1}{N}{\left(X-\hat{X}\right)}^{T}(X-\hat{X}))+\frac{2P}{N}$$

where $\hat{X}$ denotes the estimated expression profile of the target protein, i.e. $\hat{X}$ = *φ *· $\hat{\theta }$. After the statistical selection of *P *parameters by minimizing the AIC, we can determine whether the protein interaction is significant or a false positive.

In our results, '1' represents protein *i *interacting with protein *j *if the interaction is within *P *significant interactions, and '0' represents protein *i *not interacting with protein *j *if the interaction has less than *P *significant interactions. It must be considered that protein-protein interaction networks represent mutual binding relationships; if protein *i *binds to protein *j*, then protein *j *also binds to protein *i*. To determine mutual relationships in the high false positive yeast-two-hybrid experiments, we use the Boolean logical 'AND' to determine the interaction maps. Two proteins were considered to interact only if each protein binds to the other protein (see Supplementary Table 1). If an interaction is detected only in HeLa cells but not in normal cells, we call this a 'gain-of-function'; if the interaction is detected in normal cells but not HeLa cells, we call it a 'loss-of-function'. Table 2 shows the truth table of all 16 events in the sample space of comparisons of individual protein-protein interactions between HeLa cells and normal cells, as determined with our AIC detection algorithm. Protein complexes are only considered once, because of incomplete information from online databases. We iteratively refined interactions in the initial network using a similar procedure. Finally, this yielded a refined protein-protein interaction network for HeLa and normal cells.

**Table 2:** Truth table of all 16 events in the sample space of comparisons of individual interaction of protein *i *and protein *j *between HeLa and normal cells

AIC detection results in HeLa cells			AIC detection results in normal cells			Comparisons between HeLa and normal cells
		
Target protein *i*	Target protein *j*	'AND' results	Target protein *i*	Target protein *j*	'AND' results	
0	0	0	0	0	0	
0	0	0	0	1	0	
0	0	0	1	0	0	
0	0	0	1	1	1	Loss-of-function
0	1	0	0	0	0	
0	1	0	0	1	0	
0	1	0	1	0	0	
0	1	0	1	1	1	Loss-of-function
1	0	0	0	0	0	
1	0	0	0	1	0	
1	0	0	1	0	0	
1	0	0	1	1	1	Loss-of-function
1	1	1	0	0	0	Gain-of-function
1	1	1	0	1	0	Gain-of-function
1	1	1	1	0	0	Gain-of-function
1	1	1	1	1	1	

We provide all Matlab programs and codes of these figures drawn by Osprey 1.2.0 [60] in 'Additional file 9' and 'Additional file 10'. In the Matlab programs, we consider the target protein BAX as an example. Readers can simulate other target proteins using a similar procedure.

## Authors' contributions

LHC performed simulations, evaluated the results and wrote the paper. BSC provided the topic, direction and some suggestions.

## Supplementary Material

Additional file 1 **Supplementary Table 1**. Global protein-protein interactions of apoptosis in HeLa cells and normal primary human lung fibroblasts. Click here for file

Additional file 2 **Supplementary Table 2**. Gain-of-function proteins in Figure 2A, ranked by the degree of perturbation. Click here for file

Additional file 3 **Supplementary Table 3**. Loss-of-function proteins in Figure 2B, ranked by the degree of perturbation. Click here for file

Additional file 4 **Supplementary Table 4**. Eighty-six BCL2-interacting proteins identified from our results, the HPRD (Human Protein Reference Database), and literature review. Click here for file

Additional file 5 **Supplementary Table 5**. Dynamic caspase protein-protein interactions in HeLa cells. Click here for file

Additional file 6 **Supplementary Table 6**. Dynamic caspase protein-protein interactions in normal human primary lung fibroblasts. Click here for file

Additional file 7 **Supplementary Table 7**. Microarray data [26] containing genomic expression profiles of HeLa cells under ER stress. Click here for file

Additional file 8 **Supplementary Table 8**. Microarray data [26] containing genomic expression profiles of normal human primary lung fibroblasts under ER stress. Click here for file

Additional file 9 **Matlab Programs**. Five Matlab programs were used in this study. These include the AIC method, protein-protein interaction networks of apoptosis under ER stress in HeLa cells and normal primary human lung fibroblasts, and dynamic protein-protein interaction networks of caspase activation under ER stress in HeLa and normal cells. Click here for file

Additional file 10 **Codes of Figures**. Eight Osprey codes used to drawing the Figures showing protein-protein interactions in this study (Figs. 1, 2, and 4). Click here for file
